# Inhibitors of the integrase–transportin-SR2 interaction block HIV nuclear import

**DOI:** 10.1186/s12977-018-0389-2

**Published:** 2018-01-12

**Authors:** Jonas Demeulemeester, Jolien Blokken, Stéphanie De Houwer, Lieve Dirix, Hugo Klaassen, Arnaud Marchand, Patrick Chaltin, Frauke Christ, Zeger Debyser

**Affiliations:** 1 Laboratory for Molecular Virology and Gene Therapy, Department of Pharmaceutical and Pharmacological Sciences, KU Leuven, Kapucijnenvoer 33, VCTB +5, Bus 7001, 3000 Leuven, Flanders Belgium; 2 Center for Innovation and Stimulation of Drug Discovery (CISTIM), Leuven, Belgium; 3 Center for Drug Design and Development (CD3), KU Leuven R&D, Leuven, Belgium; 4 Present Address: The Francis Crick Institute, London, UK

**Keywords:** Drug discovery, Integrase, Transportin-SR2, HIV, Nuclear import

## Abstract

**Background:**

Combination antiretroviral therapy efficiently suppresses HIV replication in infected patients, transforming HIV/AIDS into a chronic disease. Viral resistance does develop however, especially under suboptimal treatment conditions such as poor adherence. As a consequence, continued exploration of novel targets is paramount to identify novel antivirals that do not suffer from cross-resistance with existing drugs. One new promising class of targets are HIV protein–cofactor interactions. Transportin-SR2 (TRN-SR2) is a β-karyopherin that was recently identified as an HIV-1 cofactor. It has been implicated in nuclear import of the viral pre-integration complex and was confirmed as a direct binding partner of HIV-1 integrase (IN). Nevertheless, consensus on its mechanism of action is yet to be reached.

**Results:**

Here we describe the development and use of an AlphaScreen-based high-throughput screening cascade for small molecule inhibitors of the HIV-1 IN–TRN-SR2 interaction. False positives and nonspecific protein–protein interaction inhibitors were eliminated through different counterscreens. We identified and confirmed 2 active compound series from an initial screen of 25,608 small molecules. These compounds significantly reduced nuclear import of fluorescently labeled HIV particles.

**Conclusions:**

Alphascreen-based high-throughput screening can allow the identification of compounds representing a novel class of HIV inhibitors. These results corroborate the role of the IN–TRN-SR2 interaction in nuclear import. These compounds represent the first in class small molecule inhibitors of HIV-1 nuclear import.

## Background

Control of human immunodeficiency virus type 1 (HIV-1) infection still poses considerable challenges. Viral resistance selection under suboptimal treatment conditions together with long-term adverse effects of chronic combination antiretroviral therapy (cART), highlight the need for novel antivirals with (1) a higher barrier towards resistance development, (2) pharmacokinetic properties allowing once-daily dosing and (3) less adverse effects. Novel targets, both viral and cellular, therefore need to be evaluated for their potential to be targeted with a small molecule in order to develop new classes of antivirals. HIV is characterized by its ability to stably integrate into the host cell genome and to infect both dividing and non-dividing cells [1]. As a result, the pre-integration complex (PIC) needs to be actively transported across the nuclear envelope. Integration into host cell chromatin is a complex process catalyzed by the viral integrase (IN). Once integration has been achieved, the fate of virus and host cell are irreversibly linked, highlighting this step as the point-of-no-return in the viral replication cycle.

Due to technical issues such as poor in vitro solubility, IN was last of the three HIV enzymes to be targeted by cART. To date, three IN inhibitors have been approved for clinical use (raltegravir, elvitegravir and dolutegravir), all belonging to a class of catalytic site inhibitors known as integrase strand transfer inhibitors (INSTIs, see [2] for a recent review). As a consequence of their common mechanism of action these inhibitors suffer a significant degree of cross-resistance. Allosteric inhibitors potently blocking IN indirectly can circumvent cross-resistance in analogy to non-nucleoside reverse transcriptase inhibitors (NNRTIs) [3]. Of note, successful integration and completion of the viral replication cycle in general, are dependent on more than just the catalytic activity of IN but also on its multimerization dynamics and interplay with cellular cofactors such as LEDGF/p75 [4–8]. Screening for inhibitors of either the LEDGF/p75-IN interaction or the integrase 3′-processing reaction independently led to the discovery of a second class of IN inhibitors, LEDGINs (also referred to as non-catalytic site integrase inhibitors, NCINIs) [9, 10]. By binding the LEDGF/p75 binding site across the IN dimerization interface, LEDGINs not only block LEDGF/p75 binding but also perturb IN multimerization, which in turn leads to inhibited catalytic activity (both 3′processing and strand transfer) and aberrant viral maturation [4–7, 11, 12].

In 2008, another IN cofactor was identified, Transportin-SR2 (TRN-SR2, Tnpo3), encoded by the *TNPO3* gene. TRN-SR2 was picked up as a cellular cofactor of HIV-1 in two genome-wide siRNA screens [13, 14] and as a binding partner of HIV IN in a yeast two-hybrid screen [15]. Through q-PCR analysis and the use of a cellular nuclear import assay [16], Christ et al. [15] showed a clear reduction in HIV nuclear import after depletion of TRN-SR2, supporting a role of TRN-SR2 in this process. Transportin-SR2 belongs to the β-karyopherin family. It has been shown to import splicing factors to the nucleus, most of which contain an RS (arginine–serine) repeat region and/or an RNA recognition motif (RRM) domain [17–19]. Its overall toroid structure, composed of stacked HEAT repeats, provides flexibility to accommodate a variety of cellular cargoes [19–21]. Charged residues on and around an Arg-rich helix in TRN-SR2 are critical for recognition of the phosphorylated RS region of cargo and hence its nuclear import [19]. Until now, crystal structures of TRN-SR2 alone [19], in complex with RanGTP [21] and in complex with the cellular cargo ASF/SF2 [19] have been described. A crystal structure of TRN-SR2 in complex with IN is not available.

A variety of viral components have been linked to nuclear import of the HIV pre-integration complex (PIC): capsid (CA), the central polypurine tract (cPPT), IN, matrix and viral protein R [22–24]. Also for the host cell, a plethora of import factors have been implicated, most notably importin-α/β [25, 26], importin-α3 [27] and importin-7 [28]. Despite the general agreement on the importance of TRN-SR2 for HIV nuclear import, the exact mechanism of action remains a matter of debate. The TRN-SR2–CA interaction has been reported to play a role in nuclear import by some groups [29, 30], while others published evidence for a direct interaction with HIV IN [15, 31–33]. Moreover, IN was shown to be displaced from TRN-SR2 upon addition of RanGTP, as is the case with normal cargoes [20]. An IN R263A/K264A mutant is partially deficient for the interaction with TRN-SR2 [33, 34] and the corresponding virus was affected at the nuclear import step, supporting the notion that the IN–TRN-SR2 interaction is responsible for this process [34].

As evidenced by the discovery and development of LEDGINs, targeting protein–protein interactions between IN and cellular cofactors can yield new classes of viral replication inhibitors [35, 36]. Since nuclear import represents a bottleneck during HIV replication [15] we reasoned that inhibitors of this interaction might have the potential to become potent antivirals and we embarked on a drug discovery campaign targeting the interaction between HIV-1 IN and TRN-SR2. Small molecules disrupting the interaction and blocking nuclear import would additionally be valuable to study HIV nuclear import and therefore increase our understanding of this crucial step in its replication cycle.

At the time this study was initiated, the interface between TRN-SR2 and IN had not been defined and no crystal structure of TRN-SR2 was available. Therefore, we opted for a high-throughput screening (HTS) approach. Here, we describe the development and use of an amplified luminescent proximity homogenous assay (AlphaScreen)-based screening cascade to identify small-molecule inhibitors of the HIV-1 IN–TRN-SR2 interaction from a library of 25,608 compounds. We eliminated false positives and nonspecific protein–protein interaction inhibitors through the implementation of appropriate counterscreens. Five compound classes provided modest protection against HIV-1 during multiple round replication. Finally, four representative compounds were tested in a cellular fluorescent HIV nuclear import assay. Two compounds significantly reduced the number of nuclear PICs, suggesting these molecules represent a novel class of inhibitors targeting HIV nuclear import. These novel inhibitors validate the IN–TRN-SR2 interaction as an antiviral target and warrant further exploration.

## Methods

### Recombinant protein purification

Recombinant proteins were expressed in *E. coli* strain BL21-CodonPlus2 (DE3). N-terminally His_6_-tagged and untagged IN, His_6_-tagged glutathione-S-transferase (GST-His_6_), maltose-binding protein (MBP)-tagged JPO2 (Cell division cycle-associated 7-like protein, CDCA7L), 3xflag-tagged LEDGF/p75 and GST-TRN-SR2 were purified as described previously [20, 37–39].

### High-throughput screening

Compound stocks were dissolved at approximately 5 mM in dimethylsulfoxide (DMSO) in 96-well plates. Stocks were first diluted to 125 µM in assay buffer (150 mM NaCl, 25 mM Tris–HCl pH 7.4, 1 mM MgCl_2_, 0.1% (v/v) Tween-20 and 0.1% (w/v) bovine serum albumin (BSA)) supplemented with 2.5% (v/v) DMSO before 10 µl was transferred to a dry 384-well PS 384-OptiPlate (Perkin Elmer) on a Freedom EVO200 liquid handling robot (Tecan). Untagged TRN-SR2 at 2.5 µM and assay buffer containing 5% DMSO were transferred from a separate 96-well plate to the assay plates and represented the positive and negative controls, respectively. Next, 5 µl each of 5× working dilutions of GST-TRN-SR2 and His_6_-IN were added using an XRD-384 automated reagent dispenser (FluidX). The plate was left to incubate for 1 h at 7 °C before 5 µl of a glutathione donor and Ni^2+^-chelate acceptor AlphaScreen bead mixture (Perkin Elmer) was added. This brought the final assay volume to 25 µl and established final concentrations of 50 µM for the compounds, 10 nM GST-TRN-SR2, 40 nM His_6_-IN, 10 µg/ml donor and acceptor beads and 2% DMSO.

### Hit validation and counterscreens

Both the hit validation assay and the two counterscreens (GST-His_6_ and LEDGF/p75–JPO2) were performed in duplicate. Compounds were first diluted to 125 µM as during the main screen but now two doses were transferred to a dry assay plate. From this point on, the hit validation assay was performed identically to the main screen.

For the GST-His_6_ counterscreen, 10 µl of a 2.5× working dilution of the recombinant GST-His_6_ protein (10 nM final assay concentration) was added to the plate instead of the GST-TRN-SR2 and His_6_-IN. In this case, 50 µM bromophenol blue and buffer containing the appropriate amount of DMSO represented the positive and negative controls, respectively.

The LEDGF/p75–JPO2 specificity counterscreen was performed similarly, but instead of GST-TRN-SR2 and His_6_-IN, maltose-binding protein (MBP)-tagged JPO2 and 3xflag-tagged LEDGF/p75 were employed at final concentrations of 5 nM. The AlphaScreen bead mixture was modified accordingly to anti-flag acceptor beads and streptavidin donor beads coated with anti-MBP antibody according to the manufacturer’s protocol (Perkin Elmer). Untagged LEDGF/p75 at a final concentration of 1 µM and buffer containing the appropriate amount of DMSO were used as positive and negative controls, respectively.

### Multiple round antiviral activity assay

The inhibitory effect of antiviral compounds on the HIV-induced cytopathic effect (CPE) in MT-4 cell culture was determined by the MTT-assay [40]. The assay is based on the reduction of the yellow colored 3-(4,5-dimethylthiazol-2-yl)-2,5- diphenyltetrazolium bromide (MTT) by mitochondrial dehydrogenase of metabolically active cells to a blue formazan derivative, which can be measured spectrophotometrically. The 50% cell culture infective dose of the HIV strain was determined by titration of the virus stock on MT-4 cells. For the antiviral activity assays, MT-4 cells were infected with 100–300× 50% cell culture infective doses in the presence of five-fold serial dilutions of the compounds. The concentration achieving 50% protection against the HIV CPE (the 50% effective concentration, EC_50_), was determined as well as the concentration killing 50% of the MT-4 cells (the 50% cytotoxic concentration, CC_50_).

### Single round antiviral activity assay

20,000 HeLaP4 cells were seeded into 96-well plates on the day prior to infection. Cells were infected in triplicate with 3 dilutions (typically 1 × 10^5^, 3.3 × 10^4^ and 1.1 × 10^4^ pg p24) of VSV-G pseudotyped single-round HIV-1 supplemented with one of the compounds in a total volume of 200 µl per well. The virus was produced as described previously [31]. The final compound concentration in the assay was 100 µM. p24 measurements were performed with the Innotest HIV Antigen mAb kit (Fujirebio). 24 h after infection the supernatant was replaced by fresh medium. 72 h post infection cells were lysed in 50 µl of lysis buffer (50 mM Tris/HCl, pH 7.3, 200 mM NaCl, 0.2% NP40, 5% glycerol) and analyzed for firefly luciferase activity (ONE-Glo™, Promega, Belgium) according to the manufacturer’s protocol. Chemiluminescence was measured with a Glomax luminometer (Promega, Belgium). The signals were normalized for protein content as determined by a BCA protein assay (Thermo Scientific Pierce).

### PIC nuclear import assay

The PIC nuclear import assay was performed as previously described [34]. Briefly, to produce the fluorescent HIV particles, 293T cells were transfected with 15 µg pVpr-IN-eGFP, 15 µg pNL4-3.Luc.R-.E- (obtained from the NIH AIDS Reference and Reagent Program), and 5 µg of the pMD.G plasmid encoding the vesicular stomatitis virus glycoprotein (VSV-G) [41]. Supernatant was collected after 48 h, filtered and concentrated by ultracentrifugation. p24 was measured with the Innotest HIV Antigen mAb kit (Fujirebio). A viral inoculum of 3.10^6^ pg p24 was used to infect 30,000 HeLaP4 cells in the presence of 100 µM of hit compound. PF-3450074 (PF74, Sigma-Aldrich) was tested at 2 µM and 10 µM. Five hours after infection, cells were fixed and the nuclear lamina was visualized with a monoclonal anti-lamin A/C antibody (Santa Cruz, sc-7292) followed by a secondary goat anti-mouse IgG Alexa-Fluor 633. Three-dimensional image stacks were acquired on a Zeiss LSM510 multiphoton confocal microscope (Cell Imaging Core CIC, University of Leuven) equipped with a Plan-Apochromat 63×/1.4 Oil DIC objective. The Z-step size was 0.3 µm. The quantification of PICs was performed using a homemade MatLab routine (The MathWorks, Inc.). A fluorescent spot was assigned as a PIC if at least two adjacent pixels were above the threshold and if the signal was present in at least two consecutive Z-planes. PICs were classified as cytoplasmic or nuclear based on the nuclear lamin staining.

## Results

### Optimization of the IN–TRN-SR2 AlphaScreen

**Fig. 1:**
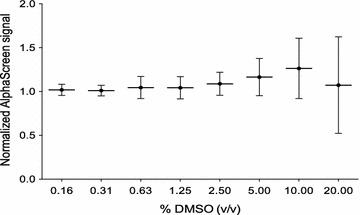
Tolerance for dimethylsulfoxide (DMSO) in the AlphaScreen assay. DMSO was titrated out starting from 20% (v/v) in the IN-TRN-SR2 interaction assay and the resulting AlphaScreen signal was measured. Results are normalized to the 0% DMSO condition

Development of robust high-throughput assays requires optimal buffer conditions in which both proteins and the detection method are stable and reproducible. Initial conditions for an AlphaScreen assay measuring the direct protein–protein interaction between HIV-1 IN and TRN-SR2 were previously reported by our group [31]. This experience allowed us to readily adapt an assay buffer to meet the requirements for a high-throughput screen (HTS) for inhibitors of the interaction. Two modifications were made to the previously reported assay buffer: First, measurements were done in the presence of 1 mM dithiothreitol (DTT) to maintain a sufficiently reducing environment and prevent aggregation of glutathione-S-transferase (GST)-tagged protein on the surface of the glutathione donor beads [39]. Second, since protein–protein interactions can be difficult to disrupt by small molecules, we aimed to screen at a relatively high compound concentration of 50 µM. To aid compound solubility at these concentrations, aside from the already present 0.1% (v/v) Tween-20 and 0.1% (w/v) bovine serum albumin (BSA), we increased the final concentration of dimethylsulfoxide (DMSO). To assess the tolerance of the assay for DMSO, we titrated it out in a two-fold dilution series between 20 and 0.16% (v/v). The AlphaScreen signal was normalized to the 0% DMSO condition and the background signal (100 and 0%, respectively). Figure 1 shows that despite a high tolerance of the assay for DMSO the variability increases to unacceptable levels when more than 2.5% DMSO is present. Based on these results, we opted for a final DMSO concentration in the assay buffer of 2%.

HTS assay optimization is a fine balance between identifying robust assay conditions with minimal variability and keeping costs acceptable. In the present case, costs were mainly driven by the amount of AlphaScreen beads used. However, reducing the bead concentration leads to a roughly linear decrease of the signal-to-background (S/B) ratio, and hence of the assay quality as well [39]. As a compromise, we decided to lower the final bead concentrations only two-fold to 10 µg/ml.

**Fig. 2:**
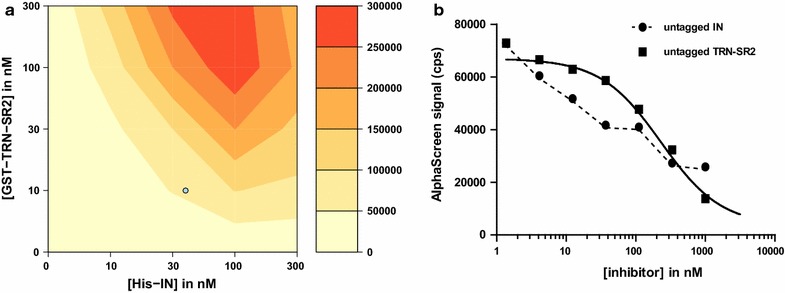
Cross-titration of His_6_-IN and GST-TRN-SR2 and evaluation of positive controls. **a** Both proteins were titrated against one another and the AlphaScreen signal measured (from yellow/low to red/high signal). The optimal condition containing 10 nM GST-TRN-SR2 and 40 nM His_6_-IN is indicated with a blue circle. GST, glutathione S-transferase; IN, integrase. **b** Untagged TRN-SR2 and IN were titrated out in the IN–TRN-SR2 interaction assay. Untagged IN was unable to reduce the signal to background levels most likely due to its ability to multimerize. Untagged TRN-SR2 could block the interaction completely and was selected as positive control

Next, optimal protein concentrations were determined through cross-titration of the binding partners (Fig. 2a). Concentrations of 10 nM GST-TRN-SR2 and 40 nM His_6_-IN provided a good S/B ratio (> 25) with minimal protein consumption and remained well below the AlphaScreen hooking range. Hooking can be seen to occur at concentrations of 300 nM His_6_-IN. Under these conditions, the Ni^2+^-chelate acceptor beads are expected to be fully saturated and the excess of free IN protein will compete with IN on the bead surface for binding to TRN-SR2, effectively inhibiting the signal.

To monitor per plate quality, each 384-well plate contained a total of 64 control wells arranged in 2 columns of 8 positive and 8 negative wells on either side. As a positive control, we evaluated both untagged TRN-SR2 and IN to corroborate the ability of our assay to pick up inhibitors. However, untagged IN proved unable to inhibit the AlphaScreen signal to background levels (Fig. 2b), most likely due to the proneness of the enzyme to form multimers at higher concentrations [42, 43] which could still bring both beads together. Untagged TRN-SR2 did inhibit the signal (Fig. 2b) and was chosen as the positive control at a final concentration of 1 µM.

### High-throughput screening, false positives and negatives

**Fig. 3:**
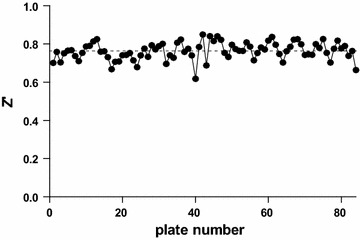
Assay performance throughout the screening campaign. Assay performance per plate was monitored throughout the screening campaign. A median Z’-factor of 0.76 was determined. No plates had to be repeated

The optimized assay was used to screen a part of the CD3 (Center for Drug Design and Discovery) library. The set of test compounds consisted of 25,608 small molecules which were selected based on (1) chemical diversity, (2) druglike properties (Lipinski rule of five compliant), (3) exclusion of known toxicophores and purchased from multiple commercial suppliers. Assay performance was evaluated on a plate-by-plate basis and remained robust throughout the entire screening campaign (Fig. 3). A median Z′-factor of 0.76 was obtained and no plates failed during screening. Considering a cut-off of 50% inhibition (percentage of inhibition (PIN) of 50%), we identified a total of 409 initial hits from the collection.

**Fig. 4:**
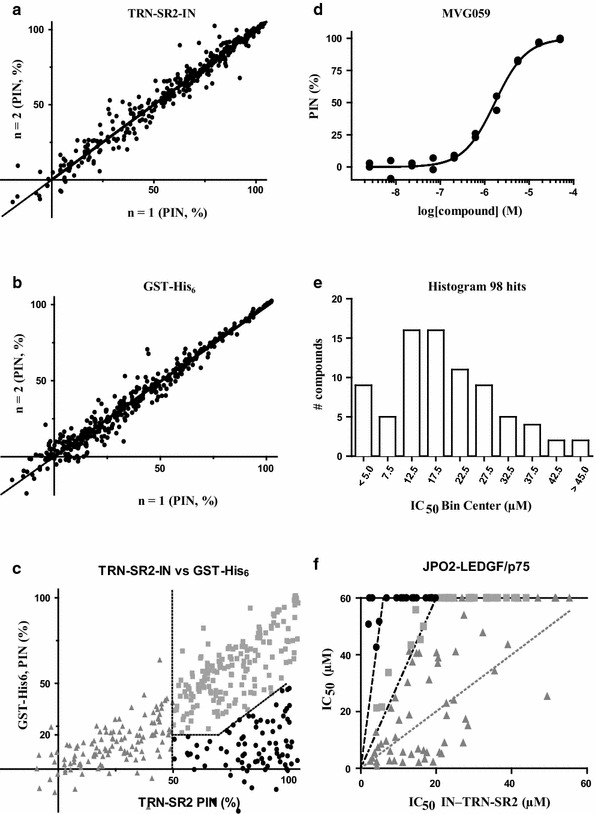
AlphaScreen screening strategy (**a, b**). Normalized percentage inhibition (PIN) of duplicates of each of the 409 primary hits in (**a**) a confirmatory repeat of the IN–TRN-SR2 AlphaScreen or (**b**) the GST-His_6_ false positive counterscreen. In both cases, the linear regression fit to the data is shown: slopes are not significantly different from 1 and R^2^ values are 0.9675 and 0.9640 for (**a**) and (**b**), respectively. **c** Average percentage inhibition in the GST-His_6_ counterscreen plotted against that observed in the IN–TRN-SR2 assay. Hits of interest show > 50% inhibition of the IN–TRN-SR2 interaction and are devoid of quenching at the active concentration (< 20% inhibition of the GST-His_6_ signal or 50% stronger inhibition of the IN–TRN-SR2 interaction). Primary hits occupying the different areas of the result space are plotted as dark grey triangles (false positives), light grey squares (quenchers) and black circles (hits of interest). **d** Titration results and dose–response curve fit obtained for compound MVG059. Duplicates are plotted. **e** Histogram of the obtained IC_50_ values for all 98 hits sorted into 5 µM bins. **f** Hit compound specificity. IC_50_ values obtained against the IN–TRN-SR2 and LEDGF/p75–JPO2 interactions were plotted against one another. Compounds having an IC_50_ < 20 µM against the IN–TRN-SR2 interaction and being tenfold less active or having an IC_50_ > 60 µM against LEDGF/p75–JPO2 are preferred (black circles). Hits that were only between 10 and 3 times more active in the IN–TRN-SR2 assay are less promising (light grey squares). Those that were less potent (IC_50_ ≥ 20 µM) but did not inhibit the LEDGF/p75–JPO2 interaction (IC_50_ > 60 µM) were looked at case-by-case. Compounds falling outside of these regions were considered non-specific and not investigated further (dark grey triangles)

False positives often dominate initial hit lists obtained from HTS campaigns. True false positives were weeded out from the cherry-picked hit compounds by a confirmation screen in which the 409 hits were retested in duplicate in the IN–TRN-SR2 interaction assay. Figure 4a shows percentages of inhibition (PIN) of the duplicates of each compound normalized to the positive (100%) and negative (0%) controls. Linear regression results in a slope of 1.000 ± 0.004 and an R^2^ of 0.9675, indicating the robustness of the platform.

AlphaScreen technology is not insensitive to interference [44]. To rid our hit lists of AlphaScreen-specific false positives including inner filter effect, ^1^ΔO_2_ quenchers, and Ni^2+^ chelators, we included in our cascade a GST-His_6_ counterscreen described previously [39]. Figure 4b shows PIN values for the duplicates of each compound in this counterscreen normalized to the positive (bromophenol blue, inner filter effect, 100%) and negative (0%) controls. Here as well, the slope of 0.9892 ± 0.006 and R^2^ of 0.9640 confirm the platform’s robustness.

Combining both results by plotting the average PIN in the GST-His_6_ counterscreen against that from the confirmation screen allowed us to confidently select positive hits (Fig. 4c). First, we opted for compounds that met the cut-off of 50% inhibition of the IN–TRN-SR2 interaction, eliminating 126 false positives. Second, compounds should preferentially be devoid of quenching at the active concentrations. Specifically, the percentage of inhibition in the GST-His_6_ counterscreen should be below 20% or the activity observed against the IN–TRN-SR2 interaction should be > 50% stronger than the quenching. These requirements delineated an area of the results space containing 98 clean and confirmed hit compounds (Fig. 4c, black circles).

### Determination of potency and specificity

The 98 confirmed hits were next subjected to IC_50_ determination in the IN–TRN-SR2 AlphaScreen interaction assay. Compounds were titrated in duplicate in a three-fold dilution series starting at 50 µM and dose–response curves were fit to the resulting data. Figure 4d shows a representative titration result while Fig. 4e provides a histogram of the obtained IC_50_ data. In total, 14 out of the 98 hits had an IC_50_ value in the single digit micromolar range.

Since the IN–TRN-SR2 protein–protein interface is believed to be relatively flat and featureless, we decided to perform a specificity counterscreen. Compounds were assayed for their inhibition of an unrelated protein–protein interaction (LEDGF/p75–JPO2) [45, 46] and their IC_50_ values were determined. Compounds were titrated as before and the inhibition of the interaction between full-length 3xflag-tagged LEDGF/p75 and MBP-tagged JPO2 was measured by AlphaScreen. Dose–response curves were fit and the resulting IC_50_ values compared to those obtained for the IN–TRN-SR2 interaction. Compounds that showed high activity (IC_50_ < 20 µM) in the IN–TRN-SR2 interaction assay and were tenfold less active or completely inactive (IC_50_ > 60 µM) against the LEDGF/p75–JPO2 interaction were prioritized (Fig. 4f). Compounds that were between 10 and 3 times more active in the IN–TRN-SR2 assay were kept as back-up compounds. Those that were less potent (IC_50_ ≥ 20 µM) but did not inhibit the LEDGF/p75–JPO2 interaction (IC_50_ > 60 µM) were looked at case-by-case and evaluated as potential back-up compounds based on their PIN at the highest concentration used. All compounds falling outside these regions of the results space were considered nonspecific protein–protein interaction inhibitors and discarded from the hit list. In the end, this resulted in 23 first priority and 25 fallback compounds.

### Analogues

The 23 first priority compounds were clustered into 12 classes based on their structure and the hit compounds of each cluster were repurchased. Unfortunately, the IN–TRN-SR2 activity could not be confirmed for 5 hits which left us with 7 hit compounds for which commercially available analogues were selected and ordered. These analogues were funneled through the same screening cascade consisting of the IN–TRN-SR2 interaction assay and both counterscreens (GST-His_6_ for quenching and LEDGF/p75–JPO2 for specificity) leading to a selection of compounds which were further evaluated for inhibition of HIV-1 replication and cellular toxicity in the MTT/MT-4 viral replication assay (see Table 1).

**Table 1:** Antiviral activity and toxicity of selected hits in multiple round MTT assay

Class	Compound ID	IN–TRN-SR2 Pin at 50 µM (%)^a^	EC_50_ (µM)^b^		CC_50_ (µM)^c^		Protection (%)^d^
3	MVG001	65.3	<	8	<	8	2
	MVG002	43.4	>	13	=	13	5
	MVG003	47.2	>	80	=	80	13
	MVG004	82.6	>	21	=	21	4
	MVG005	39.7	>	57	=	57	6
	MVG006	50.5	>	63	=	63	6
	MVG007	64.8	>	74	=	74	6
	MVG008	30.1	>	99	=	99	23
	MVG009	32.0	>	51	=	51	4
6	MVG010	37.1	>	100	=	100	6
	MVG011	41.7	>	102	=	102	1
	MVG012	107.1	>	74	=	74	10
	MVG013	51.9	>	95	=	95	3
	MVG014	63.8	>	87	=	87	4
	MVG015	48.1	>	87	=	87	5
	MVG016	46.9	>	244	>	244	20
	MVG017	< 10	=	195	=	229	52
	MVG018	< 10	>	137	>	137	7
	MVG019	< 10	>	250	>	250	6
	MVG020	< 10	>	250	>	250	18
	MVG021	< 10	>	250	>	250	34
	MVG022	44.9	=	67	>	136	53
	MVG023	< 10	>	110	>	110	0
	MVG024	< 10	>	36	>	45	49
7	MVG025	72.9	>	34	=	34	4
	MVG026	45.7	>	35	=	48	4
	MVG027	43.2	>	57	=	57	6
	MVG028	58.7	>	56	=	80	13
	MVG029	48.8	>	57	=	57	3
	MVG030	71.6	>	97	=	97	25
	MVG031	62.3	>	53	=	53	4
	MVG032	46.5	>	48	=	48	5
	MVG033	49.5	>	59	=	59	20
	MVG034	43.0	>	73	=	73	16
9	MVG035	43.8	>	36	=	36	1
	MVG036	105.6	>	55	=	65	46
	MVG037	43.1	>	85	=	85	22
	MVG038	47.2	>	59	=	59	0
	MVG039	40.1	>	17	=	17	1
	MVG040	43.6	>	25	=	25	25
	MVG041	54.0	>	35	=	35	2
10	MVG042	89.6	>	55	=	55	10
	MVG043	102.0	>	67	=	67	4
	MVG044	75.9	>	250	>	250	22
	MVG045	11.4	>	203	=	203	21
	MVG046	10.3	>	207	=	207	32
	MVG047	< 10	>	250	>	250	7
	MVG048	< 10	>	87	=	87	0
	MVG049	< 10	=	165	>	231	55
	MVG050	44.1	>	250	>	250	0
	MVG051	< 10	>	250	>	250	22
	MVG052	20.1	>	66	=	66	8
	MVG053	< 10	>	167	>	167	39
	MVG054	18.0	>	225	=	225	0
	MVG055	< 10	>	167	>	167	21
	MVG056	71.1	>	250	>	250	24
	MVG057	23.3	>	250	>	250	16
	MVG058	53.7	=	170	>	250	24

^a^Compounds were tested in AlphaScreen at 50 µM for their ability to inhibit the IN-TRN-SR2 interaction. Percentage inhibition (PIN), relative to the DMSO control

^b,c^MT-4 cells were infected with HIV at 100 to 300× the 50% cell culture infective doses in the presence of 100 µM of the antiviral drugs. The compound concentration achieving 50% protection against the cytopathic effect of HIV, the 50% effective concentration (EC_50_), was determined. The concentration of the compound killing 50% of the MT-4 cells, the 50% cytotoxic concentration (CC_50_), was determined as well

^d^The percentage of protection against the cytopathic effect of HIV was measured in MT-4 cells as well. Values below 20% were not considered as real activities. Data are averages of triplicate measurements

### Antiviral activity in cell culture

A selection of active compounds was then evaluated for inhibition of HIV-1 replication and cellular toxicity in the MTT/MT-4 viral replication assay. The percentage protection against the HIV CPE was measured. Modest (up to 46% at 100 µM) protection was observed for a limited number of compounds in classes 3, 7 and 9 but no 50% effective concentration (EC_50_) was reached at concentrations lower than the observed 50% cytotoxic concentration (CC_50_) (Table 1).

**Fig. 5:**
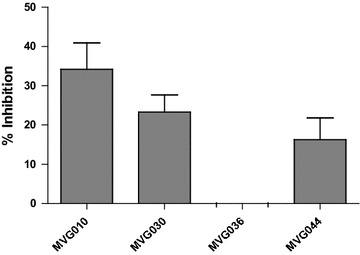
Representative hit compounds inhibit single round viral replication. HeLaP4 cells were infected with 3.3 × 10^4^ pg p24 of a single-round virus expressing the firefly luciferase reporter gene (Fluc) in the presence of 100 µM of one of the compounds. After 72 h, cells were lysed and the percentage inhibition of HIV replication compared to the DMSO control was measured. Mean and standard deviation of two independent experiments, each performed in triplicate, are presented

Rapid growth (and frequent cell divisions) of the MT-4 cells during the viral replication assay however, may mask viral nuclear import defects, in particular with low potency compounds. This could imply that the MTT/MT-4 assay is not well suited to detect a block in viral nuclear import. We hence decided to evaluate four representative compounds from the most promising classes 6, 7, 9 and 10 in a single round antiviral activity assay on HeLaP4 cells. Compounds were tested at a final concentration of 100 µM and cells were infected with a threefold dilution series of a single-round virus expressing the firefly luciferase reporter gene (Fluc). The percentage inhibition of HIV replication compared to the DMSO control was determined. For compound MVG036 no inhibition could be detected while MVG010, MVG044 and MVG030 showed 34.2, 16.3 and 23.3% inhibition, respectively (Fig. 5).

### Effect on HIV nuclear import

**Fig. 6:**
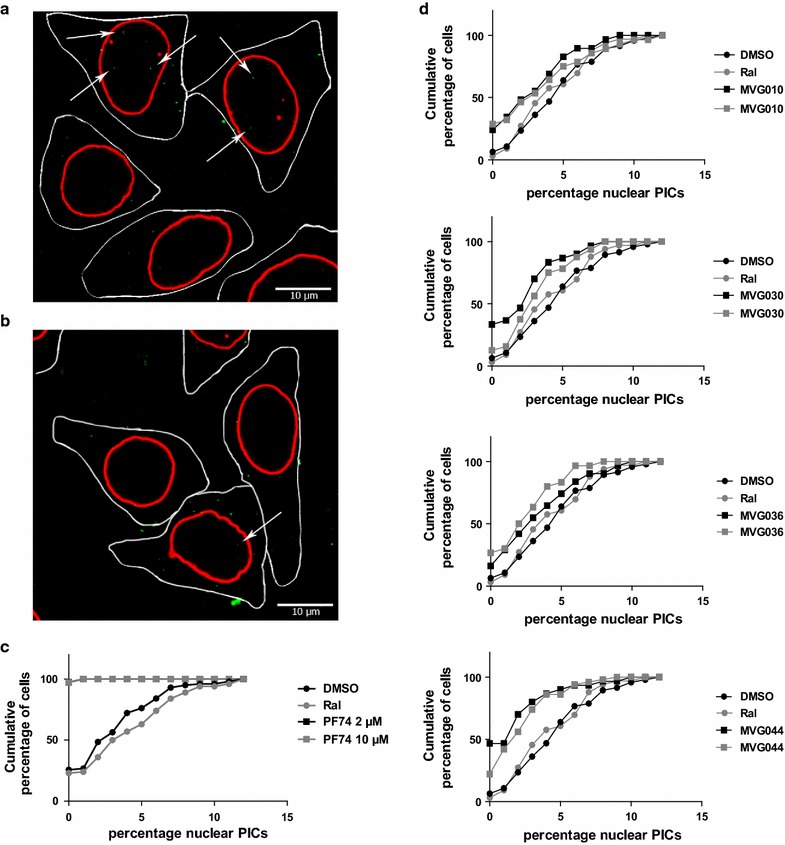
Representative hit compounds reduce nuclear import of HIV. Five hours after infection with IN-eGFP labeled virus in the presence of one of four representative hit compounds (MVG010, MVG044, MVG030 or MVG036), PF74 and DMSO or RAL as controls, HeLaP4 cells were fixed and analyzed by laser-scanning confocal microscopy. The ratio of nuclear/total amount of PICs was quantified (percentage nuclear PICs). **a, b** Representative slice of a stack of cells infected with eGFP-IN labeled virus in the presence of DMSO (**a**) or MVG044 (b). The nuclear lamina was immunostained with anti-lamin a/c (red). PICs are identified as green dots and nuclear PICs are highlighted by white arrows. **c, d** Presented are the cumulative distributions of the percentage of cells containing the indicated percentage nuclear PICs for the positive control PF74 (**c**) and four representative hit compounds (MVG010, MVG044, MVG030 or MVG036) or DMSO and Ral as controls (**d**). Two independent experiments, with distinct virus productions, are presented for each compound

We next analyzed the selected compounds, again at 100 µM concentration, in a low throughput PIC nuclear import assay [34] to test whether the decrease in viral replication is due to inhibition of PIC nuclear import. In this assay fluorescently labeled viral PICs are visualized using confocal microscopy and their subcellular localization is evaluated based on a nuclear lamina staining (Fig. 6a, b). The ratio of nuclear PICs over the total number of PICs (percentage nuclear PICs) was then calculated as a measure of nuclear import. DMSO and Raltegravir (RAL) were used as negative controls in two independent experiments as they should not affect HIV nuclear import. The capsid-binder PF74 was used as a positive control. This compound is known to inhibit nuclear import at low concentrations (2 µM) whereas it also inhibits reverse transcription at higher concentrations (10 µM) (Fig. 6c) [47, 48]. MVG044 and MVG030 significantly reduced the number of nuclear PICs compared to both DMSO and RAL (*p* < 0.05) while MVG036 only showed significance compared to DMSO (Fig. 6d; Table 2).

**Table 2:** PIC assay statistics

	Compared to DMSO				Compared to Raltegravir			
	*n* ^a^	*p* value^b^	% nuclear PICs^c^	95% CI^d^	*n* ^a^	*p* value^b^	% nuclear PICs^c^	95% CI^d^
DMSO		–	–	–	49	0.8099	4.8	[3.8; 5.9]
Ral	35	0.8099	4.5	[3.4; 5.6]		–	–	–
MVG010	32	0.1209	3.8	[2.3; 5.4]	29	0.1152	4.2	[2.2; 6.3]
MVG044	31	0.00001	2.1	[1.5; 2.7]	50	0.0002	2.0	[0.9; 3.2]
MVG030	30	0.0411	3.3	[2.3; 4.2]	33	0.0019	2.2	[1.4; 3.0]
MVG036	31	0.0011	2.3	[1.6; 3.1]	30	0.0700	3.2	[2.2; 4.2]

Summary of two independent PIC assays comparing the four representative hit compounds to the DMSO and Ral control. HeLaP4 cells were fixed and analyzed by confocal microscopy 5 h after infection with an eGFP-labeled IN virus. PICs were assigned as cytoplasmic or nuclear based on nuclear lamina staining

^a^Number of HeLaP4 cells counted in each condition

^b^*p* values (Mann–Whitney *U* test) compared to DMSO or RAL control

^c^Mean percentage of nuclear PICs in each cell calculated using a homemade MatLab routine (The MathWorks, Inc.)

^d^95% confidence interval (CI) for the percentage of nuclear PICs

MVG044 from compound family 10 induced the most pronounced block to nuclear import: the mean  % nuclear PICs was 4.8% for DMSO (95% confidence interval [3.8; 5.9%]), 4.5% for Ral [3.4; 5.6%] and 2.1% [0.9; 3.2%] and 2.0% [1.5; 2.7%] for the two MVG044 conditions (DMSO, n = 49 cells; MVG044, n = 50 cells; *p* = 0.00001, Mann–Whitney *U* test and Ral, n = 35 cells; MVG044, n = 31 cells; *p* = 0.0002, Mann–Whitney *U* test) (Table 2).

In Table 3 we present an overview of the data for the four representative compounds. At first sight, there does not seem to be a strong correlation between the different read outs. This is not entirely unexpected, mainly due to the modest activity of our compounds. In addition, these compounds are products of commercially available libraries and have not been optimized yet for activity. The experiments on the potency determination in MT4 cells as well as the inhibition of viral infectivity in a single round viral replication assay in HeLaP4 cells, were experiments lasting for 5 and 3 days, respectively. Chemical (in)stability of the compounds might lead to a more pronounced effect when early time points are analyzed (PIC assay) in contrast to analysis after incubation over several days.

**Table 3:** Overview of the data for four representative compounds

	In vitro		MT4 cells			HeLaP4 cells	
	AlphaScreen^a^		MTT^b^			Fluc^c^	PIC assay^d^
	IC_50_ (µM)	PIN (%)	EC_50_ (µM)	CC_50_ (µM)	% Protection	PIN (%)	*p* value
MVG010	54.6	37.1	> 100	100	6	34.2	0.1209
MVG030	33.6	71.6	> 97	97	25	23.3	0.0411
MVG036	37.6	105.6	> 55	65	46	–	0.0011
MVG044	17.1	75.9	> 250	> 250	22	16.3	0.00001

^a^HIV-1 IN–TRN-SR2 AlphaScreen IC_50_ values and percentage inhibition (PIN) are presented. All compounds were tested at 50 µM

^b^EC_50_ and CC_50_ values in MT4 cells are given as well as the percentage of protection against the cytopathic effect of HIV

^c^HeLaP4 cells were infected with a single-round virus expressing the firefly luciferase reporter gene (Fluc) in the presence of one of the compounds (100 µM) and the results are expressed as a percentage inhibition relative to the DMSO control

^d^For the PIC assay in HeLaP4 cells, *p* values (Mann–Whitney *U* test), compared to the DMSO control, are given

## Discussion

Here we present the discovery of small HIV-1 nuclear import inhibitors targeting the interaction of HIV-1 IN and its cellular co-factor, the karyopherin TRN-SR2. We developed an AlphaScreen-based HTS assay to screen for inhibitors of the HIV IN–TRN-SR2 interaction (Figs. 1, 2, 3). We screened a diverse library of 25,608 compounds, yielding a total of 409 hits (Fig. 4). A confirmation assay on the cherry-picked hits in combination with a GST-His_6_ counterscreen to remove AlphaScreen technology-interfering false positives narrowed down the hit list to 98 inhibitors (Fig. 4c). Of these 98 hits, 14 were found to have an IC_50_ value in the single-digit micromolar range as determined in the TRN-SR2–HIV IN AlphaScreen assay (Fig. 4e). Because we anticipated the IN–TRN-SR2 interface to be relatively flat and featureless, we performed an additional specificity counterscreen for an unrelated protein–protein interaction (LEDGF/p75–JPO2). Selecting compounds that did not inhibit the LEDGF/p75–JPO2 interaction or were 10 times more potent against the IN–TRN-SR2 interaction resulted in a list of 23 first priority compounds (Fig. 4f). After clustering, hit confirmation with a new “fresh” sample, and analogs selection, the best compounds selected from 6 series were evaluated for their ability to block HIV replication in infected cells. For five of the classes we were able to detect modest protection against the HIV CPE in an MTT/MT-4 antiviral activity assay (Table 1). In a next step, compounds from the most promising classes 6, 7, 9 and 10 were tested in a single-round antiviral activity assay and a HIV nuclear import assay on HeLaP4 cells. Although only modest inhibition of viral replication could be detected (Fig. 5), two of the compounds significantly reduced the number of nuclear PICs while the other two showed a trend towards less nuclear import (Fig. 6; Table 3).

As previously mentioned, no information on the structure and interface of the TRN-SR2–HIV IN complex was available at the start of our screening campaign. In 2012, De Houwer et al. identified the interaction hot spots in IN for the IN–TRN-SR2 interaction [32]. Amino acids R262/R263/K264 and K266/R269 in the C-terminal domain of IN were found to be the main determinants of the interaction. These results were independently confirmed by Larue et al. [33]. More recently, the structures of TRN-SR2 alone and its complexes with Ran-GTP and ASF/SF2 were published [19, 21]. Although the structure of TRN-SR2–IN has not yet been solved, structural information together with the mutagenesis data point towards a large, charged and relatively flat interaction interface. Targeting the TRN-SR2–IN interaction with high affinity may therefore require design of completely new chemotypes [49].

## Conclusions

Our efforts evidenced that an Alphascreen-based HTS can allow the identification of compounds representing a novel class of HIV inhibitors. Their activity in the PIC nuclear import assay confirms that nuclear import of HIV can be targeted by small molecules. While the effects are clear-cut in the PIC assay, the activities of the compounds in the MTT/MT-4 and the single round antiviral activity assay are only modest (Table 3). Several reasons may be conceived to explain this discrepancy. First, rapid growth (and frequent cell divisions) of the cell culture-adapted MT-4 cells during the viral replication assay may relieve the bottleneck of nuclear import. Disassembly and reassembly of the nuclear membrane during the frequent mitoses could give incoming viral PICs periodic access to the condensed, LEDGF/p75-decorated chromatin, masking nuclear import defects. Second, while the single round antiviral activity assay gives a global view of viral replication after 72 h, the PIC assay is a kinetic assay, providing a snapshot of the population of cytoplasmic and nuclear PICs at 5 h after infection. As nuclear import is a bottleneck process [15], PICs accumulating at the nuclear periphery may remain there for some time before being bound and imported by TRN-SR2 (or being degraded). Depending on its affinity and kinetics, an inhibitor of the IN–TRN-SR2 interaction could delay the import process and shift PICs towards degradation in the cytoplasm. Our compounds may not induce sufficient delay in import and a few PICs may make it into the cell’s nucleus masking the inhibition.

One of the caveats of targeting pathogen-host protein–protein interactions is the risk of inducing cellular toxicity due to inhibition of the host protein function. Notably, none of the active classes exhibited prominent toxicity in the MTT/MT-4 assay.

Recently, De Houwer et al. [34] reported an IN mutant virus that is partially defective for interaction with TRN-SR2 and nuclear import. Together with these findings, the identification of molecules inhibiting HIV-1 PIC nuclear import from a HTS campaign against the IN–TRN-SR2 interaction underscores the importance of the interaction for HIV-1 nuclear import.
